# “I am putting my fear on them subconsciously”: a qualitative study of contraceptive care in the context of abortion bans in the U.S.

**DOI:** 10.1186/s12978-024-01908-9

**Published:** 2024-11-24

**Authors:** Yasaman Zia, Erica Somerson, Connie Folse, Alejandra Alvarez, Kathryn Albergate Davis, Alison B. Comfort, Katherine Brown, Kristyn Brandi, Ghazaleh Moayedi, Cynthia C. Harper

**Affiliations:** 1 Department of Obstetrics, Gynecology, and Reproductive Sciences, Bixby Center for Global Reproductive Health, University of California San Francsico, San Francisco, CA USA; 2 Ryan Residency Training Program, University of California San Francisco, San Francisco, CA USA; 3 Pegasus Health Justice Center, Dallas, TX USA; 4 Philip R. Lee Institute for Health Policy Studies, University of California, San Francisco, San Francisco, CA USA

## Abstract

**Background:**

Since the *Dobbs vs. Jackson Women’s Health Organization* decision in June 2022, providers throughout the U.S. have been navigating the shifting legal landscape of abortion bans, which diminish the delivery of evidence-based healthcare. The *Dobb*s decision has had a detrimental impact on medical training, the physician–patient relationship, and provision of medical care. However, few studies have captured the effects on providers in adjacent fields, including contraceptive care. Our objective was to examine the impact of *Dobbs* on contraceptive care.

**Methods:**

We conducted semi-structured in-depth interviews (August 2022–July 2024), with 41 contraceptive healthcare providers across the US, with the majority (63%) in abortion restrictive states. We utilized deductive thematic analysis to assess providers’ practice changes and experiences related to contraceptive services.

**Results:**

In reaction to the *Dobbs* decision, providers noted increased requests for contraception, especially for highly effective methods. Providers worried that certain methods, such as IUDs or emergency contraception, would become restricted, and mentioned advance provision of pills and other ways that they would try to ensure supplies. Providers also discussed that their patients were worried about threats to contraception, including for adolescents. Some expressed concern, however, that the abortion bans may prompt providers to overemphasize high-efficacy methods with directive counseling. Providers shared that it was stressful to practice in contexts of uncertainty, with shifting abortion policies affecting contraceptive care, including emergent needs such as providing contraceptive services to out-of-state patients before they go home. Several providers shared that they felt an increased importance of their role in their communities, and a deepened commitment to advocate for their patients.

**Conclusions:**

Abortion restrictions profoundly impact providers’ contraceptive counseling and care. The effects of *Dobbs* on providers and their clinical practices underscore providers' legally precarious position in today’s reproductive health landscape. Attention to contraceptive access and person-centered care has become a salient public health need across the U.S. The long-term impacts of limited reproductive rights may stretch an already under-resourced healthcare system and further emphasize moral pressures.

## Plain Language Summary

Since the *Dobbs vs. Jackson Women’s Health Organization* decision in June 2022, providers throughout the U.S. have been navigating the shifting legal landscape of abortion bans, which diminish the delivery of evidence-based healthcare. The *Dobbs* decision has had detrimental impacts on health care, including medical training, the physician–patient relationship, and how medical care is provided for patients. This study is focused on the impacts of the *Dobbs* decision on the clinic practices and experiences of providers in contraceptive care, where patients receive birth control counseling and methods. We conducted interviews with healthcare providers working in contraceptive care to assess their practice changes and personal reactions since the *Dobbs* decision. Providers noted changes in increased patient requests for certain contraceptive methods, uncertainty about abortion restrictions affecting contraceptive care, and feeling personally obligated to provide contraceptive care and medical advice around abortion despite the shifting legal landscape. Providers also expressed personal experiences of stress, or moral injury, around the high stakes impact of abortion restrictions on patients as well as confusion and uncertainty of how legal restraints impact their work. Nevertheless, they felt an increased emphasis on the importance of their roles in their communities. Abortion restrictions profoundly impact providers’ contraceptive counseling and care. Providers’ comments underscored a sense of moral injury, a feeling of urgency to help patients, and shifts in counseling approaches that may over-emphasize contraceptives in the context of limited abortion access.

## Introduction

The *Dobbs vs. Jackson Women’s Health Organization* ruling in June 2022 profoundly impacted reproductive healthcare provision throughout the US. Abortion bans have had the most direct impact on patients, as evidenced by research stressing the stark increases in the number of patients traveling across state lines for abortion care, the hardships of navigating the financial and logistical barriers to access care, and the compounding systemic inequities that marginalized communities experience in the healthcare system [1–8]. While abortion care has been most directly affected, as shown in a growing body of research [9–13], the overreach of the *Dobbs* decision has extended into many facets of reproductive health. Several recent studies have assessed the detrimental impact of *Dobbs* on pregnancy care, the physician–patient relationship, and medical education, though less research has explored the impact on healthcare providers in other affected areas, including contraceptive care [10–13]. Healthcare providers working in abortion care are experiencing moral injury, defined in this context as high-stakes action or inaction that are a violation of one’s moral code and may cause life-altering outcomes for patients [9, 14, 15]. The ambiguous and uncertain landscape of reproductive health care since the *Dobbs* ruling places a psychosocial strain on providers [9]. Some evidence suggests these pressures may impact clinical judgement by either over-complying (i.e. restricting clinical practice above and beyond what is required by the law) due to uncertainty about changing policies or conscientiously committing to providing abortion care in spite of legal restraints [11, 16–18].

Previous research on gynecologists who had provided abortion care in restrictive states found a wide range of perceived impacts of the *Dobbs* ruling, including that it posed an occupational hazard for these clinicians and, at the same time, a maternal health crisis [9]. However, the *Dobbs* ruling likely has impacts that have reverberated throughout the US healthcare system, including for in-vitro fertilization in some states. With sharp increases in travel to other states for abortion care, several studies have established the strain on the healthcare system [16, 19–23]. There are compelling reasons that the *Dobbs* decision affects other areas of reproductive health care now that access to abortion care has become even more limited, especially contraceptive services that prevent unwanted pregnancies and are often provided alongside abortion services. Yet very little research has examined the adverse effects of this ruling on these services. Since contraception and abortion may exist on a spectrum of services to prevent and manage undesired pregnancies, abortion restrictions may set a precedent for potential future restrictions on contraception. We aim to expand upon the scope of the *Dobbs* impact among a wider range of healthcare providers—specifically those providing contraceptive care. The objective of this study is to examine the impact of *Dobbs* on providers’ experiences as well as practice changes in contraceptive care drawing on evidence we gathered from contraceptive care providers across both abortion restrictive and non-restrictive states.

## Materials and methods

We conducted semi-structured in-depth interviews with healthcare providers to evaluate healthcare professionals’ experiences in contraceptive care in the US with a focus on the consequences of the *Dobbs* ruling. We recruited clinicians and educators (referred to herein as healthcare providers) attending University of California, San Francisco Continuing Medical Education-accredited online and in-person trainings on contraception. After the trainings, we invited all attendees across several training sessions occurring from August 2022 to July 2024 by emailing invitations. The inclusion criteria limited the sample to healthcare providers who currently offered contraceptive education or care themselves and were located in the US. We specifically recruited from trainings reaching participants from across different regions of the US. A total of 114 training attendees were interested in participating in the study, of which 111 were eligible (healthcare providers working in contraception and located in the US), 57 completed the pre-interview survey, and 16 were no shows or cancellations. We enrolled a total of 41 healthcare providers, including 11 physicians, 22 advance practice clinicians, 5 nurses, and 3 counselors working in contraceptive care.

We developed a semi-structured interview guide to cover emerging topics in healthcare including experiences in care provision since the *Dobbs* ruling, as examined in this analysis. Other general topics included in the interview guide were transgender healthcare, discrimination and bias, and telehealth. After obtaining verbal consent, we conducted interviews virtually via Zoom. All interviews were audio-recorded and lasted approximately 45–60 min. The research team members who conducted the interviews (AA, ES, KAD) identified as Latinx, Jewish, and White cis-gender women. Participants were compensated with a $250 gift card.

We transcribed recordings verbatim. We developed a codebook based on both deductive and inductive methods. The deductive codes were based on topic areas that were included in the structured interview guide (practice changes and providers’ experiences post-*Dobbs*) while the inductive codes synthesized participants’ responses. The inductive codes were generating by reading 10 interviews and broadening the codebook to fully capture the concepts raised in conversation. The team coded two interviews together to become familiar with the codebook, and reconciled differences in coding methodology through iterative discussion. Once we established a similar approach to applying codes, two researchers (ES, YZ) individually coded each interview transcription. The two coders identified as cis-gender women of Jewish and Middle Eastern backgrounds (ES, YZ). All coding was completed in Atlas.ti Version 9.1.7.0 (Berlin, Germany). We utilized a deductive thematic analysis to assess providers’ reflections and changes to their practice after the *Dobbs* decision. We coded the state where providers practiced as either abortion restrictive or non-restrictive, per Guttmacher’s abortion restrictions map, to protect their identities [24].

The Institutional Review Board at University of California, San Francisco approved the study (#12-10336).

## Results

The study sample included 41 healthcare providers in 18 states and Washington, DC, with the majority (63%) in abortion restrictive states (Table 1). We present themes in two main domains related to the impact of Dobbs on contraception provision: changes to practice and providers’ experiences. Within changes to practice, main themes were: changes in contraceptive method requests and counseling practices; risk avoidance; and conscientious provision (i.e., personal obligation to providing contraception and medical advice related to abortion). Within providers’ experiences, main themes were: moral injury (i.e., due to the high-stakes impacts of abortion restrictions on patients and threats to other care); confusion of how legal restrictions impact their work; the contemplation of threats to contraceptive care and that certain methods might become unavailable; and an increased emphasis on their roles in their communities.

**Table 1:** Overall participant characteristics (N = 41)

	N	%
*Provider type*		
Physician	11	27%
Advance practice clinician	22	54%
Nurse practitioner	18	44%
Physician assistant	3	7%
Nurse midwife	1	2%
Nurse	5	12%
Counselors	3	7%
*State*		
CA (non-restrictive)	5	12%
DC (non-restrictive)	1	2%
FL (restrictive)	1	2%
GA (restrictive)	2	5%
IL (non-restrictive)	1	2%
IN (restrictive)	1	2%
LA (restrictive)	1	2%
MN (non-restrictive)	2	5%
MO (restrictive)	1	2%
NC (restrictive)	2	5%
NJ (non-restrictive)	1	2%
NM (non-restrictive)	1	2%
NY (non-restrictive)	3	7%
OK (restrictive)	4	10%
OR (non-restrictive)	1	2%
TN (restrictive)	8	20%
TX (restrictive)	5	12%
VA (restrictive)	1	2%
*Abortion-restrictive state*		
Yes	26	63%
No	15	37%

### Changes to practice

*Changes in method requests and counseling practices* Many providers noted an increased volume of requests for long-acting reversible contraceptive methods (LARCs) among patients after *Dobbs* as well as for permanent methods*.* While much of the discussion centered on provision of methods for patients assigned female at birth, two healthcare providers noted an increase in clients seeking vasectomies post-*Dobbs*. They observed notably higher interest in this permanent method in the period following the *Dobbs* decision.

“I’ve definitely had a lot of patients come in and they’re worried that IUDs [intrauterine device] are going to be outlawed, so they want one. I see that a lot. And I’ve seen a lot of people bringing in their adolescents because they’re worried that birth control is going to be outlawed. That is the biggest fear and repercussion I’ve seen in my office, is they’re all worried about their future access to birth control so they want to get it now. That’s what I am seeing right now.” (Nurse Practitioner, restrictive state).

“I’ve also seen since that ruling more men in the last couple months come in for vasectomies than I have for 16 years.” (Physician Assistant, non-restrictive state).

“I think the most interesting piece I’ve interacted with quite a bit is people calling for vasectomies. Since the Supreme Court (SCOTUS) ruling. That is something that we actually track because we’re interested in adding that to our line of care. But vasectomies is something we’ve heard a lot more about lately.” (Social Worker, restrictive state).

A few providers in non-restrictive states noted an increase in out-of-state patients seeking injectable contraception**,** or depot-medroxyprogesterone acetate (DMPA, or “Depo”), as a method they can initiate onsite, which provides a 3-month period of contraceptive coverage when they travel back to their home state. The shift in patterns of contraceptive choices following *Dobbs* highlights the gaps in contraceptive care that exist in certain states that have low funding for reproductive healthcare, which may have become more salient for patients.

“I feel like I’ve had a lot more people want Depo ... and I don’t really have a good explanation for why. It tends to be just a lot of the out of state patients who are like I don’t know what birth control I want. I don’t want to have to think about it for a couple months, so just give me one shot today and then I will think about what I want to do moving forward.” (Nurse Midwife, non-restrictive state).

One provider also discussed the importance of offering contraceptive care for medically complex patients, which include patients with disabilities and patients for whom pregnancy would be high risk.

“We see a lot of medically complex patients being in a large children’s hospital. And knowing that for a patient with a heart transplant, pregnancy of any kind but especially an unplanned pregnancy is a really big no-no. Both for their medical, like their health, and for the fact that there are almost no options [for abortion] if they do become pregnant… is… informs my counseling as well.” (Physician, restrictive state).

The heightened awareness of the urgency of contraception and increased request for long-acting methods also created pressure on counseling around LARCs. Several providers worried about the impact of increased pressure on patients and potential coercion of contraceptive counseling within their own approaches.

“I do think that providers and pediatricians in particular feel like everybody needs a LARC right now basically. And so I do worry that those conversations that they’re having are a little bit more like directive you know. A little bit more subtly coercive in the direction of more LARC.” (Physician, non-restrictive state).

“I am still continuing to counsel them, but I do find myself feeling a little bit more fearful when a patient does leave without birth control and they don’t want to get pregnant. Being that I can’t counsel them on anything related to that topic [of abortion] legally. That’s definitely been hard too, because we also see patients for pregnancy testing here. So that’s something that we interact with quite a bit. I don’t know that it’s changed my counseling practices in general, but I feel like it’s changed the way I feel about birth control in that it’s allowed me to feel a little bit more biased because I am putting my fear on them subconsciously I think.” (Nurse Practitioner, restrictive state).

*Risk avoidance*. As state-level abortion restrictions were passed, providers experienced confusion on how the scope of legal restrictions extended into contraceptive services. Providers described changes to their contraceptive practices, more than was required, as a result of restrictions to abortion care. Some providers shared examples of clinical decision-making that suggested over-compliance as excessive avoidance of risk related to new legal restrictions on abortion care. Other providers expressed worry that they may be seen as interrupting a pregnancy in placing an IUD.

“Sometimes you use cytotec misoprostol before an IUD insertion to soften the cervix and make it more comfortable to place. And then we’re just like is someone going to think we’re causing an abortion. Is someone going to try to get us in trouble for doing that? Is the pharmacy going to fill it? We know we’re not doing anything wrong, but... that too.” (Nurse Practitioner, restrictive state).

“Our state, we were one of the trigger states that immediately banned pretty much all abortions of any kind. And so... I do feel like I am a little more stressed and worried about repercussions or that I am going to do the wrong thing or get in trouble for putting in an IUD. There are some doctors that I am working with who have started doing SDHCG [serum detected human chorionic gonadotropin test] instead of just a urine HCG to prove that they weren’t pregnant before they put in an IUD and those kinds of things.” (Nurse Practitioner, restrictive state).

*Conscientious provision.* While legal restrictions on reproductive health following *Dobbs* were focused largely on abortion care, providers anticipated potential additional restrictions extending to contraceptive care. Numerous healthcare providers implied their willingness to stock emergency contraception and oral contraceptive pills for advance provision or to find contraceptives over the border in Mexico, where it is available without prescription, to bring back to the US. This is an example of conscientious provision, defined as providers conscientiously committing to providing care in spite of legal restraints [17].

“I feel very fortunate that we’re in [Western state]. I feel like we’re in a little bubble over here. But I feel a responsibility, I listen to a lot of podcasts about what women are doing around the country for mailing these birth control pills. I’ve thought about my proximity to Mexico and access there. I’ve gone through a lot of ways where I like told my fiancé I am going to go to Mexico and get a bunch of pills and start mailing. He’s like slow down, I am like yeah you’re right.” (Nurse Practitioner, non-restrictive state).

“I keep requesting it [emergency contraception (EC)]. One time we went to like a street festival this summer and we brought it. And some people stocked, they like took 20 of them. I don’t care, take them it doesn’t matter to me. But we definitely always try to have it on hand. Especially people traveling back and if people are scared to travel back with it, we’ll take it out of the packaging. And say no one needs to know what this little pill is.” (Social Worker, non-restrictive state).

In addition to the rippling effects on contraceptives, providers expressed concern regarding the full range of reproductive counseling and care, including abortion. When placed in the precarious position of not being able to provide the full range of reproductive health care due to legal restrictions, some providers emphasized their preference for conscientiously providing medical advice regarding abortion in face of shifting legal grounds. One provider also mentioned the importance of safe medical care for patients.

“I wanted to make it clear that our clinic was a clinic that we would do whatever we needed to do to help her with her decision. It didn’t feel like it was a good encounter to me, she [a pregnant patient] was... going out of state and it was kind of unclear where she was going. And I tried to like get the information about if she was going somewhere safe to be doing this procedure. And it just felt very difficult, especially I work in a red state and I felt like it was very hard for me to even establish this medical safety of what she was getting done which was my priority.” (Registered Nurse, restrictive state).

### Providers’ experiences

*Moral injury*. In reaction to the *Dobbs* decision, providers expressed pervasive feelings of fear and moral injury due to their inability to provide evidence-based reproductive health care. Providers reported that they felt alarmed about the repercussions of providing reproductive health care and the potential consequences of abortion restrictions on their licenses and perceived ability to counsel patients on the full range of reproductive health options. Providers described themselves with generalized feelings of hurt, anger, and fear with not being able to serve their patients. Their fears about not being able to provide evidence-based abortion care appeared to expand with looming threats to contraceptive care, such as IUD insertion or emergency contraception.

“I feel limited. I feel hurt. I feel powerless. I feel angry with the decision. I feel like I am, I can’t advocate for the patients I want to advocate for. I feel like I have to… be very careful with the care I provide and the words I say and what I document.” (Physician, restrictive state).

“I would say maybe fear and guilt. Fear of if I do something to help out in those other states, will I get in trouble. And then guilt of what I’ve ended up doing is really not much.” (Nurse Practitioner, non-restrictive state).

#### Fear of future restrictions on contraception

Providers in restrictive states were also cognizant of the impact that the contraceptive care they offer can have on their patients and their lives. They expressed urgency about whether they would be able to offer methods like emergency contraception and IUDs in the future to patients. Within this uncertainty of whether methods would be restricted in the near future, the precariousness of contraceptive services for adolescent patients was mentioned several times as well.

“We also offer emergency contraceptive pills at our clinic. … But I definitely have concerns moving forward, is this something that will continue? Will restrictions become more and more [expanded to other sexual health care] you know? I feel a larger sense of purpose currently realizing that since that has happened in a lot of states, we are the last line of defense for these people, our last line of hope I guess. So being able to offer family planning services where they can plan on their timeline is super important.” (Nurse Practitioner, restrictive state).

#### Confusion and uncertainty

Given the shifting legal landscape, healthcare providers reported that they felt confusion and uncertainty around how to deliver contraceptive care as part of comprehensive reproductive care visits, which may include abortion. This sentiment was expressed in navigating how to provide abortion counseling and care within their clinics and across state lines. Frequent changes and ambiguity around what forms of care or medical advice would be illegal places healthcare providers in a precarious position of either not being able to provide person-centered reproductive care or placing themselves at legal risk.

“I think what’s hard is I don’t think we have a really good sense legally what we’re allowed to do now. we haven’t really gotten clarification from our institution. It’s kind of just silent. … which has been difficult. So I think we all really don’t know… what we can and cannot do.” (Physician, restrictive state).

“They’re changing those laws so fast that it’s hard to keep up with as a provider, so I can only imagine how hard it is to keep up with as an individual who might be considering those services.” (Registered Nurse, restrictive state).

Providers who deliver contraceptive care also often provide abortion counseling or care. In response to the uncertainty of legal restrictions on abortion care, and in the absence of an institutional response stemming from a lack of clarity of legal implications on clinical protocols, providers in both restrictive and non-restrictive sates reported expanding resources and referral networks for pregnant patients. Providers in abortion restrictive states attempted to find ways to connect patients to safe abortion care in other states.

“That prompted me, the next day I was looking up online, okay where can I send them? Is there a hub of information, safety resources, hotlines, things I could be giving out because I wasn’t getting that from my clinic…. I think I feel a little bit more prepared because I’ve prepared resources and also I always try to make it clear that my priority is their medical safety and I am going to support them in whatever decision they make, but I just want to make sure that they’re safe. But would I say I feel fully confident? No. Just because I feel like things are so up in the air.” (Registered Nurse, restrictive state).

“As far as caring for patients I think it’s going to be important for us to educate ourselves about the law in different states so we can offer them... while I know this is what we can do for you here, in this state you can do XYZ. Learning resources to provide them I think is going to be important going forward.” (Nurse Practitioner, restrictive state).

With the surmounting uncertainty and confusion around how abortion restrictions have impacted abortion counseling and contraceptive care, one provider noted that rise of misinformation among both patients and providers.

“There’s a lot of things now, and there’s a lot of nebulousness around it, especially for providers and laws and telehealth stuff… I talked about patient misinformation, providers give each other a lot of misinformation all the time too. So I’ve heard a lot of people say, no you can never do this, but I don’t think that’s accurate. I think there’s a lot of services that can still be very much reasonably safely provided.” (Physician, restrictive state).

*Roles in their communities*. Providers expressed an increased emphasis on the importance of their roles in their communities. They felt enhanced responsibility to serve their patients, inform them well, and advocate for them. While this theme was present across both restrictive and non-restrictive states, this was especially true for providers in non-restrictive states.

“I felt my role as a provider to be even stronger and more important in the community. It’s reaffirmed my job and my role as a healthcare provider for women, to help them be informed and make the best decision for them.” (Physician Assistant, non-restrictive state).

“But that was kind of like a catalyst to, a reminder rather... of the importance of the work that I do. Kind of like a wake up call, no this is not just a day to day job that I do. It’s important as... in a community health center specifically in an urban area, this type of work is important. And it’s not just ... day to day work, it’s important to the people that we serve. So it was kind of like a wake up call for me to keep trying harder and like advocate for my patients.” (Registered Nurse, non-restrictive state).

“I do feel a desire to work with other people around making abortion easier to access. Knowing that we may be a state that people come to from other states to seek services.” (Physician, non-restrictive state).

## Discussion

In this sample of a wide range of healthcare providers engaged in contraceptive care across both restrictive and non-restrictive states, providers noted that they experienced more requests for effective contraceptive methods, including permanent methods. An increased pressure around contraceptive access had detrimental weight on their contraceptive counseling potentially leading to an overemphasis on certain methods in the absence of abortion access. They also expressed feeling a mixture of confusion and uncertainty surrounding their clinical roles and questioning the moral expectations placed on providers in the post-*Dobbs* landscape. Providers also feared policy attacks on contraception and were concerned about patients that in general do not have high contraceptive access including adolescents. Together, these results demonstrate how *Dobb*s has undermined reproductive healthcare, including contraception, and placed extraneous stress on healthcare workers.

The proliferation of detrimental policies aimed at restricting reproductive health care services has had significant spillover effects into contraceptive counseling and care provision. As echoed by other studies, our sample of providers struggle to navigate the balance between providing person-centered contraceptive care to prevent undesired pregnancy while also avoiding the legal repercussions, namely of criminalization or revoking licensure, that may be implicated in abortion provision [25]. For example, several providers noted that the insertion of IUDs may be interpreted as a legally ambiguous contraceptive care service. Recent abortion restrictions aim to confuse the use of IUDs as emergency contraception with causing an abortion, despite the scientific evidence that its mechanism of action greatly differs from abortive properties [26]. Policy coercion, or the policies defining clinical guidelines that intentionally or unintentionally limit both provider and patient autonomy, surrounds sexual and reproductive healthcare decisions and is not limited to abortion care; it also extends into contraceptive counseling and provision. This strain effectually reduces access to the full range of contraceptive methods, since providers may be afraid to place IUDs due to concern for legal implications. The Centers for Disease Control and Prevention’s (CDC) Medical Eligibility Criteria for Contraceptive Use (MEC) recommends that providers facilitate access to the full range of contraceptive methods and provide noncoercive care to patients [27]. Not offering the full range of contraceptive methods may cause patients to perceive coercion from their provider [28].

Further, providers noted that they were concerned about IUDs and emergency contraception being the next target for these policy attacks on reproductive autonomy. The reaction of fear reverberated in approaches that may over emphasize contraceptives and lead to potential coercion in counseling sessions with patients, as found in other studies [9]. We want to emphasize, however, that contraceptive care is not a solution to a lack of policies and practices that protect abortion access. Since the passing of *Dobbs*, decreased contraceptive access and lower quality of care have been found, with the burden falling disproportionately on systemically oppressed communities, including young people, queer people, low-income people, and foreign-born people [29]. While both patients and providers may sense an increased pressure to prevent pregnancy, quality of contraceptive care is obstructed through the weaving in of potential for coercion in place of person-centered counseling and care. While efficacy is one of the many variables that patients consider when selecting contraception, the potential for directive counseling can supersede the patients wants and needs [30]. In supporting reproductive autonomy, providers must aim to maintain shared decision-making within contraceptive care practices.

Changes to contraceptive provision cited by providers in this study provide evidence of shifts in patients’ preferences and needs in reproductive healthcare since the *Dobbs* ruling. The observed increase in DMPA for out-of-state patients provides coverage for a 3-month period during which patients can reconsider their contraceptive options and access points, including user-controlled methods, such as self-injectables (sub-cutaneous DMPA). In related research, we did find a need for increased training of providers in offering sub-cutaneous DMPA for self-administration to ensure its wider availability [31]. The reports of increases in vasectomy-seeking patients in our study are also echoed by other studies, including one that found a 200% increase in vasectomy consults at a single clinic during the 6-months after the *Dobbs* ruling [32–34]. Similarly, other studies have noted an increase in tubal ligation consults since the *Dobbs* ruling [35].

Since the passing of *Dobbs*, the number of abortions in the US has essentially remained the same [7]. This underscores the importance of referral networks, abortion funds, and support for self-managed abortion that continue to facilitate abortion access in restrictive states. In our study, we found that providers often felt confusion about the legal threat of counseling patients regarding undesired pregnancies as well as a lack of support from their clinics regarding institutional policies that protect their providers and patients. We note that the lack of concerted institutional responses is reflective of disparate interpretations of the litigation processes and the shifting implications for patient care [36]. Institutions must protect their providers and equip them with tools and resources to provide person-centered care, while de-emphasizing the tendency to over-comply with legal restraints. Healthcare providers are stewards of patient care. Due to the changing nature of their jobs with regard to legal risk and decreased quality of life due to the varied impacts of legal regulations, providers may leave abortion-restrictive states [9].

The ambiguity of what may constitute the beginning of a pregnancy, an abortion, and a life-threatening pregnancy complication also creates conditions in which providers may over-comply with legal restraints—further limiting essential care [37]. As such, some providers may be worried about the ambivalence of this legal definition and, as found in this study, may be worried of being perceived as performing an abortion when providing emergency contraception. This absence of a clear definition coupled with the litigation process for enforcing newly passed restrictions have obstructed reproductive healthcare and placed an overwhelming burden on providers. Providers in other studies have cited the occupational hazard and moral injury of continuing to provide sexual healthcare in abortion restrictive states [9]. Further, different types of providers and settings of clinics may have disparate experiences of burnout and overwhelm in abortion care, and this diversity in experience may be applicable to contraceptive providers (38).

Our work had some important limitations, including the potential for selection bias for providers who responded to our calls for qualitative interviews after attending contraceptive care training provided through our program. Our sample of providers largely included advanced practice clinicians and may be different in both education and practice as compared to other types of providers. The healthcare providers that were receptive to interviews may be inherently different in their viewpoints or care provision from their colleagues who chose not to participate in research. Similarly, providers who are selecting to take part in contraceptive trainings may also be different than those not participating in these trainings, though we are unable to assess how. We did not specifically ask participants whether they provided abortion care to specify their experiences, and we did not assess the timing of *Dobbs*-related restrictions were rolled out as it relates to their interviews. Lastly, we did not systematically collect the race, gender, or practice settings of participants, which may limit the depth of our analysis on subgroups that may differentially experience the harms of *Dobbs* in clinics or in communities.

## Conclusions

Our interviews highlight the moral injury and fear among health care providers resulting from abortion bans, even in contraception care. To sustain providers, clinics must invest in training and legal support so providers can safely advocate for their patients and effectively provide healthcare in uncertain, and increasingly hostile, environments. The long-term impacts of limited reproductive rights may perpetuate coercion and reduce person-centered approaches to contraceptive counseling as well as stretch an already under-resourced healthcare system and further emphasize moral pressures.

## Data Availability

The datasets generated and/or analyzed during the current study are not publicly available due to the sensitive nature but are available from the corresponding author on reasonable request.

## Abbreviations

DMPA: Depot medroxyprogesterone acetate
EC: Emergency contraception
HCG: Human chorionic gonadotropin
IUD: Intrauterine device
LARC: Long-acting reversible contraceptive
SCOTUS: Supreme Court of the US
